# Enhanced bone cement for fixation of prosthetic joint utilizing nanoparticles

**DOI:** 10.1007/s10856-024-06848-1

**Published:** 2025-01-13

**Authors:** Safaa Gamal, Mina Mikhail, Nancy Salem, Mohamed Tarek EL-Wakad, Reda Abdelbaset

**Affiliations:** 1 Biomedical Engineering Department, Faculty of Engineering, Helwan University, Cairo, Egypt; 2 Mechatronics Engineering Department, Canadian International College, Cairo, Egypt; 3 Biomedical Engineering Department, Faculty of Engineering and Technology, Future University in Egypt, Cairo, Egypt

## Abstract

Bone cement is commonly utilized to secure prosthetic joints in the body because of its robust fixation, stability, biocompatibility, and immediate load-bearing capability. However, issues such as loosening, leakage, and insufficient bioactivity can lead to its failure. Therefore, improving its mechanical, physical, and biological properties is crucial for enhancing its efficiency. This study examines the impact of incorporating four different nanomaterials—Titanium Dioxide (TiO_2_), Magnesium Oxide (MgO), Calcium Phosphate (Ca_3_(PO_4_)_2_), and Alumina Oxide (Al_2_O_3_)—into bone cement on its mechanical, physical, and biological properties. TiO_2_ and Al_2_O_3_ nanoparticles are selected to enhance the compression strength of bone cement, thereby preventing loosening. Magnesium Oxide (MgO) and Ca_3_(PO_4_)_2_ nanoparticles are chosen to improve cell adhesion and reducing the risk of cement leakage. Five specimens were prepared: the first with 100% pure bone cement powder, the second with 98% pure bone cement powder and modified with 2% MgO and TiO2, and the remaining three with 95% pure bone cement powder and modified with 5% varying ratios of MgO, TiO_2_, Ca_3_(PO_4_)_2_, and Al_2_O_3_. Compression, tensile, hardness, and bending strengths were assessed to determine improvements in mechanical properties. Setting temperature, porosity, and degradation were measured to evaluate physical properties. Cell adhesion and toxicity tests were conducted to examine the surface structure and biological properties. The results demonstrated that the modified specimens increased compression strength by 8.14%, tensile strength by 3.4%, and bending strength by 4.96%. Porosity, degradation, and setting temperature in modified specimens increased by 3.24%, 0.64%, and 5.17% respectively pure bone cement values. Cell adhesion in modified bone cement specimens showed normal attachment when scanned with FE-SEM. All of the tested modified specimens showed no toxicity, except for specimens with 2% Al_2_O_3_ that showed 25% toxicity which could be averted by employing antibiotics.

**Graphical Abstract:**

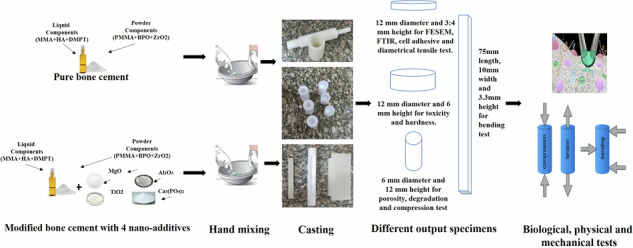

## Introduction

Recent global statistics for prosthetic joint operations show a yearly increase of up to 34% with people over 65 years recording around 498 thousand cases worldwide in the last 5 years [1]. One study predicted that prosthetic joint replacement surgeries would increase to 276% by 2030 [2]. Fixation of prosthetic joints by high-viscosity bone cement is one of the most common methods in prosthetic joint replacement operations [3, 4]. While bone cement has many benefits such as its biocompatibility, ease of handling, straightforward preparation methods, and appropriate mechanical qualities, some drawbacks also appear like cement shrinkage, leakage, and loosening after impression which can result in prosthetic joint fixation failure [5, 6]. The recurrence of fractures in people who had undergone such operations and the need to replace joints in some operations present a constant need for improving the properties of bone cement [7]. Nowadays, using additive materials with different properties added to bone cement is one of the adopted methods to enhance bone cement’s physical, mechanical, and biological properties [8, 9].

Calcium Phosphate (Ca_3_(PO_4_)_2_) as an additive material contains similar inorganic structure components of body bones, which leads to promoting body bone regeneration [10, 11]. Previous studies revealed that adding Ca_3_(PO_4_)_2_ to bone cement enhances bone defect healing and regeneration [12, 13], which may lead to good attachment and low shrinkage [14]. However, the decreasing effect of Ca_3_(PO_4_)_2_ on bone cement mechanical properties was highlighted [15]. Researchers supported using Ca_3_(PO_4_)_2_ with other additive minerals, such as Magnesium, to improve bone cement mechanical qualities by increasing the biological activity [16].

Alumina Oxide (Al_2_O_3_) is famous for its direct effect in enhancing the mechanical properties of bone cement due to its strong abrasion resistance and hardness, good biocompatibility, and stability in biological contexts [17]. It directly improves hardness and leads to decreasing surface scratches [18]. Researchers nominated using Al_2_O_3_ with high percentages in bone cement to get an enhanced composite [19]. They also discussed using a 1% additive of Al_2_O_3_ to enhance hardness and decrease the porosity of bone cement [20].

Researchers also justified using Titanium Dioxide (TiO_2_) as an additive to bone cement due to its multiple advantages such as improving elasticity, compression, and bending strengths, besides its biocompatibility [21]. Other researchers also nominated using Magnesium Oxide (MgO) with bone cement to enhance the osteoblast adhesion and bending modulus [9, 22, 23]. Ratios of 1.5% TiO_2_ w/w and 0.5% MgO are nominated to be used with bone cement to improve its mechanical and physical properties. TiO_2_ and MgO allow for enhancing compression strength by 2.8% and hardness strength by 1.89% [24].

In recent years, researchers have studied the difference in adding enhancement materials either in normal, micro, or nano size to pure bone cement. They nominated using additives in nano size to benefit from the quality of bone cement union and nano-enhanced properties of materials [25]. The targeted modified bone cement may need adding one or more ceramic, polymer, or metal nano additive materials to perform enhancement in mechanical and biological properties by combining different materials [26].

In this study, Ca_3_(PO_4_)_2_, Al_2_O_3_, TiO_2,_ and MgO are used as nano additives with varied ratios to enhance bone cement’s mechanical, physical, and biological properties. MgO and Ca_3_(PO_4_)_2_ NPs are selected to strengthen bone cement cell adhesion in order to limit cement leakage, while TiO_2_ and Al_2_O_3_ NPs are selected to increase bone cement compression strength in order to prevent loosening of bone cement in prosthetic fixation. Hardness, compression, bending, and tensile strength tests are applied to determine the enhancement of bone cement mechanical properties. Field emission scanning electron microscopy (FE-SEM) and Fourier transform infrared spectroscopy (FTIR) are used to study specimens’ morphological structure. Setting temperature, porosity, and degradation of bone cement specimens are calculated to check for the physical properties. Osteosarcoma (MG63) cells are used to check for cell adhesion and cell toxicity with the modified bone cement specimens, cell adhesion is evaluated by FE-SEM scan after 1 and 7 days of attaching the cell to bone cement, and the toxicity of modified bone cement specimens is examined using MTT (3-(4,5-dimethylthiazol-2-yl)-2,5-diphenyltetrazolium bromide) assay that depends on metabolically active cells to reduce the yellow to ensure the biocompatibility of the modified bone cement with human body.

This research presents a novel trend in combining four distinct types of nano additive materials into bone cement, where each has been previously studied separately. Each material recorded limited improvement in either mechanical or biological bone cement properties when added separately to bone cement. This study addresses these limitations by investigating new specimens with different ratios of four additive materials, providing full specifications of the mechanical properties (compression, tensile, bending, and hardness strengths), physical properties (setting temperature, porosity, and degradation) and biological properties (cell adhesion and cell toxicity) that provide several choices of bone cement properties related to patient requirements.

## Methodology

Pure high-viscosity bone cement preparation depends on the powder and liquid components ingredients. Powder components consist of Poly Methyl Methacrylate (PMMA), Benzoyl Peroxide (BPO), and Zirconia Dioxide (ZrO_2_). Liquid components consist of Methyl Methacrylate (MMA), N, N-dimethyl para-toluidine (DMPT), and Hydroquinone.

### Preparation of bone cement specimens

**Fig. 1:**
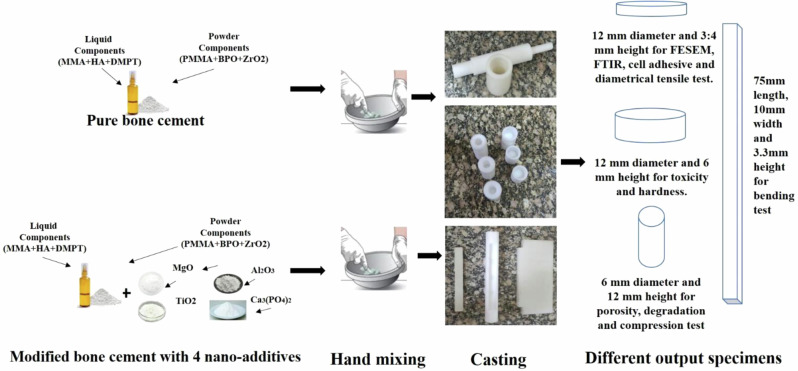
Preparation steps of pure and modified bone cement [24]

According to our previous study [24], a precise ratio of 40.85 grams of powder to 20.00 ml of liquid is recommended to generate a pure high-viscosity bone cement specimen (S_1). To prepare the proposed powder, it is recommended to use a specific component with the following percentage weights: 86.9% w/w PMMA, 0.86% w/w BPO, and 12.24% w/w ZrO2. However, the powder components that are employed have a particle size of 50 ± 20 μm. The liquid components are composed of 99.35% w/w ml MMA and 0.65% w/w ml DMPT, with Hydroquinone added at a concentration of 50 parts per million (ppm). The initial step in preparing bone cement involves thoroughly mixing the powder components on a plate with a stainless-steel tool for 30 seconds according to the recommended ratios. Simultaneously, the liquid components should be mixed in a bottle according to the recommended ratios. The second step is adding liquid to powder components and mixing well by hand for 30 seconds [27]. The preparation procedure of pure and modified bone cement is shown in Fig. 1. Four specimens’ shapes of differing diameters (shown in Fig. 1) are cast to evaluate the mechanical, physical, and biological characteristics of bone cement.

**Fig. 2:**
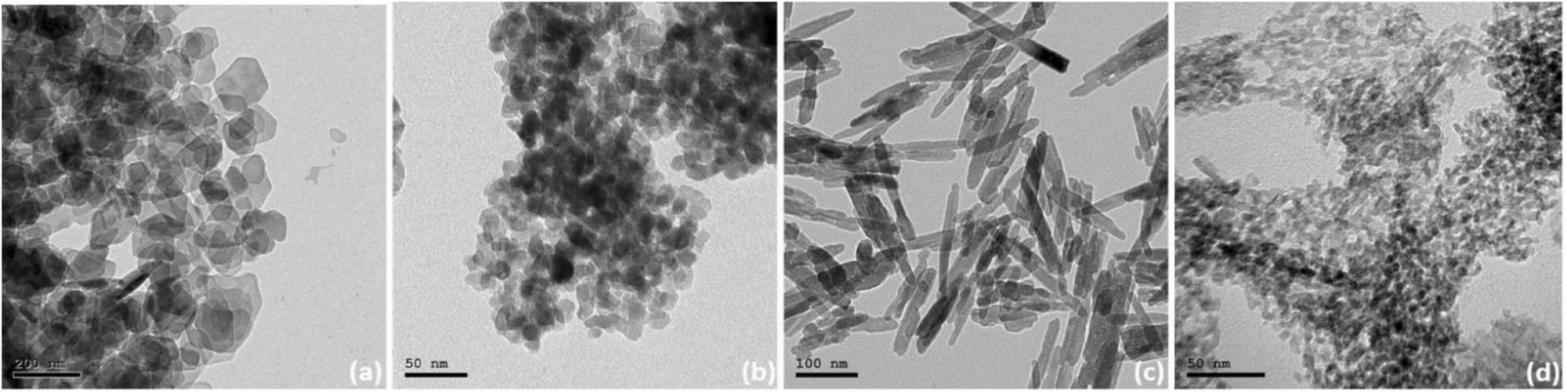
High-resolution TEM for nano additives. **a** TEM scan of MgO NPs at 200 nm, **b** TEM scan of TiO_2_ NPs at 50 nm, **c** TEM scan of Ca_3_(PO_4_)_2_ at 100 nm, and **d** TEM scan of Al_2_O_3_ NPs in 50 nm

Modified bone cement specimen S_2 consists of 98% w/w pure bone cement powder and 2% replaced by 0.5% MgO and 1.5% TiO_2_ NPs. MgO NPs are used in 20 ± 5 nm size with spherical shape as shown in Fig. 2a. MgO NPs size is checked using Transmission Electron Microscopy (TEM) with an acceleration of 200 kV and 100 nm scan magnification. TiO_2_ NPs are used in 15 ± 2 nm size with quasi-spherical shape as shown in Fig. 2b. MgO NPs size is checked using high-resolution TEM with an acceleration of 200 kV and 50 nm scan magnification. Table 1 shows the ratios of nano additive materials relative to pure bone cement components in each of the prepared five specimens.

**Table 1:** Bone cement specimen’s component ratios w/w

Sample	Pure bone cement powder	MgO NPs	TiO_2_ NPs	Al_2_O_3_ NPs	Ca_3_(PO4)_2_ NPs
**S_1**	100%	-	-	-	-
**S_2**	98%	0.5%	1.5%	-	-
**S_3**	95%	0.5%	1.5%	1%	2%
**S_4**	95%	0.5%	1.5%	2%	1%
**S_5**	95%	0.5%	1.5%	1.5%	1.5%

Modified bone cement specimens S_3, S_4, and S_5 consist of 95% w/w pure bone cement powder,2% replaced by 0.5% MgO and 1.5% TiO_2_ NPs, and 3% replaced by three different ratios of Ca_3_(PO4)_2_ and Al_2_O_3_ NPs as shown in Table 1. Ca_3_(PO_4_)_2_ NPs are used in rod shape with diameter 20 ± 5 nm and length 100 ± 30 nm as shown in Fig. 2c, Ca_3_(PO_4_)_2_ size is checked using TEM with acceleration 200 kV and 100 nm scan magnification. Al_2_O_3_ NPs are used in 20 ± 5 nm size with spherical shape as shown in Fig. 2d. Al_2_O_3_ NPs size is checked using TEM with acceleration 200 kV and 50 nm scan magnification.

### Biological and surface structure analysis

The shape and structure of bone cement specimens’ surfaces are examined using the FE-SEM instrument. FE-SEM uses samples with a diameter of 12 ± 0.1 mm and a thickness of 4 ± 2 mm coated by a gold layer to allow for polymer scanning with suitable electrostatic charging [28].

FTIR analysis is used to clarify the functional group of pure and modified specimens. Samples with diameter 12 ± 0.1 mm and thickness 6 ± 2 mm were examined at room temperature using ranges from 500 to 4000 cm^−1^ mid-IR source [29].

The biological cell culture is investigated using MG63 cell which is an established cell line of resemble osteoblast cells. The MG63 cells were added to bone cement specimens according to the American Cell Viability Test Protocol. MG63 cell line was kindly supplied by the Tissue Culture Department. Cells were maintained in MEM-E medium supplemented with 10% fetal bovine serum in a humidified atmosphere of 5% CO_2_ at 37 °C. Cells were saved according to the manufacturing protocol and were cleaned using phosphate buffer saline after the growing medium was decanted. According to the necessity, detached cells were splattered.

To investigate cell adhesion, the surface of bone cement specimens was sterilized with Ultraviolet (UV) light for 25 minutes. After that, bone cement specimens with MG63 cells were impressed in the RPMI −1640 medium. Following that, the cells were cultured and soaked for 1 and 7 days in a humidified incubator with 5% CO_2_ and 95% air environment at 37 °C. Then, cells were fixed in 4% paraformaldehyde for 4 h. followed by three washes with phosphate buffer saline (PBS) to eliminate any unattached cells and then dried with CO_2_. The dried samples were sputtered with a thin layer of gold for cell morphology observations using an electronic microscope. The cell adhesion behavior of bone cement specimens was evaluated through FE-SEM scan for MG63 cell line on the surface of bone cement specimen after 1 and 7 days [15, 28].

Usage of bone cement in human body forces using non-toxic additive materials. Cell viability or bone cement toxicity test allows for calculating and checking for the toxicity of bone cement specimens and cell viability, it also enhances the limitation of orthopedic infection that may be produced from using toxic additive materials. Cell viability was assessed by the mitochondrial-dependent reduction of yellow MTT (3-(4,5-dimethylthiazol-2-yl)-2,5-diphenyl tetrazolium bromide) to purple formazan [30, 31]. MG63 cells were suspended at 37 °C under 5% CO_2_. Cells were seeded at a concentration of 2 × 10^5^ cells/ml in fresh complete growth medium at 37 °C for 24 h under 5% CO_2_ using a water-jacketed Carbon dioxide incubator. After 48 h. of incubation medium was aspirated, 40ul MTT salt (2.5 μg/ml) was added to each specimen and incubated for a further four hours at 37 °C under 5% CO_2_. To stop the reaction and dissolve the formed crystals, 200 μL of 10% Sodium dodecyl sulfate (SDS) in deionized water was added to each specimen and incubated overnight at 37 °C. Then, the microplate reader was used to calculate the absorbance wavelength. Statistical significance was tested between samples and negative control (cells with vehicle) using an independent t-test by the SPSS 11 program. DMSO is the vehicle used for the dissolution of plant extracts, and its final concentration in the cells was less than 0.2%. The percentage of change in viability was calculated according to Eq. 1 [32, 33]:

1 $$\begin{array}{l}{\rm{Viability}}( \% )=\left({\rm{Mean}}\; {\rm{optical}}\; {\rm{density}}\; {\rm{of}}\; {\rm{extract}}\,/\,{\rm{Mean}}\;\right.\\\left. {\rm{optical}}\; {\rm{density}}\; {\rm{of}}\; {\rm{negative}}\; {\rm{control}}\right)\times 100\end{array}$$

### Physical properties analysis

Ambient temperature plays an important role in handling the characteristics of bone cement. On the other hand, the maximum temperature has a direct effect on the human tissues around the impressed bone cement. Determining the setting temperature (T_set_) depends on the maximum (T_max_) and ambient (T_amb_) temperatures as shown in Eq. 2. The setting temperature is calculated for fifteen specimens (three samples for each ratio) with 68 mm diameter and 10 ± 0.1 mm thickness according to ISO5833. A thermocouple with type E was impressed to the center of bone cement specimens to measure the temperature simultaneously with time, all of the specimens’ T_max_ was measured with T_amb_ = 23 C° [34].

2 $${T}_{{set}}=\frac{{T}_{\max }+{T}_{{amb}}}{2}$$

The porosity (P) of bone cement is marked as a vital property with values that should be kept relatively limited. Suitable porosity values allow for bone migration and bone osteoblasts, but excessive values lead to weak bone cement mechanical properties and low load-bearing capacity. Fifteen specimens (three samples for each ratio) were prepared with 6 ± 0.1 mm diameter and 10 ± 2 mm height for porosity calculation. The initial weight (W_o_) of samples is calculated after two days of preparation. All of the specimens were impressed in simulation body fluid (SBF) in an incubator at 37 °C for 28 days. Specimens’ swelling weight (W_1_) is estimated after they are removed from the SBF, washed with deionized water, and dried at 37 °C for one day. The specimens’ dry weight W_2_ is calculated after being left in air at room temperature for one day to dry [15, 20, 35–37].

Porosity is calculated according to Eq.3.

3 $$p\left( \% \right)=\frac{{W}_{2}-{\rm{Wo}}}{{W}_{2}-{W}_{1}}100 \%$$

Specimen degradation (D) is calculated by determining the dry specimen weight after being impressed for 7, 14, 21, and 28 days in SBF given the initial specimen weight [15, 36, 37]. Specimen degradation was calculated according to Eq. 4:

4 $$D\left( \% \right)=\frac{{W}_{{\rm{o}}}-{W}_{1}}{{W}_{{\rm{o}}}}100 \%$$

### Mechanical properties analysis

Micro-hardness Vickers machine is used to calculate specimen hardness value related to Eq. 5. Specimens were prepared in 12 mm diameter and 6 mm height for the hardness test [20, 38].

5 $${HV}=1.354* \frac{{\rm{F}}}{{\left(\frac{d1+d2}{2}\right)}^{2}}$$

Compression strength values are important as they give insight into the enhancement in mechanical properties of modified bone cement specimens. A Teflon mold was prepared to produce compression strength test specimens with 6 mm diameter and 12 mm height related to ISO 5833:2002 compression test dimensions. 15 specimens were prepared, 3 samples for each bone cement ratio. After 2 hours of preparation at room temperature, fracture load (F) is determined using the Universal Test Machine given the initial cross-sectional area A [34]. The compressive strength ${\sigma }_{c}$ is calculated according to Eq. 6.

6 $${\sigma }_{c}=\frac{F}{A}$$

The tensile strength of specimens is determined according to the Brazilian test. 15 specimens, 3 specimens for each ratio, were prepared with 12 mm diameter (D) and 3 mm thickness (T). The preparation time is 2 h. at room temperature. Fracture load F is determined by subjecting specimens to varying loads through the diameter of the specimen until it is broken [38]. Tensile strength ${\sigma }_{t}$ is determined according to Eq. 7.

7 $${\sigma }_{t}=\frac{2F}{\pi {DT}}$$

Bending strength is an effective key in indicating the enhancement in mechanical properties of modified bone cement specimens. A Teflon mold was prepared to produce bending and flexure strengths rectangular bar specimens with length (L) 75 mm, width (w) 10 mm, and thickness (t) 3.3 mm related to ISO 5833:2002 dimensions. After 2 h. of preparation at room temperature, 15 specimens were tested, 3 samples for each bone cement ratio [29, 39]. The fracture load (P_f_), the distance of the inner points of load (L), and the standard loading span for the three-point bend specimen (S) are calculated during the test. The bending strength ${\sigma }_{b}$ is calculated according to Eq. 8:

8 $${\sigma }_{b}=\frac{3{P}_{f}L}{w{{dt}}^{2}}$$

## Results

The enhanced properties of modified bone cement specimens are verified by checking for their biological, physical, and mechanical properties. The biological and surface structure properties are checked by FE-SEM scan and FTIR to assess cell adhesion and toxicity. Setting temperature, porosity, and degradation are checked to assess the physical properties. Mechanical properties are checked by applying some of the mechanical tests such as hardness, compression, tensile, and bending tests.

### Biological and surface structure results

**Fig. 3:**
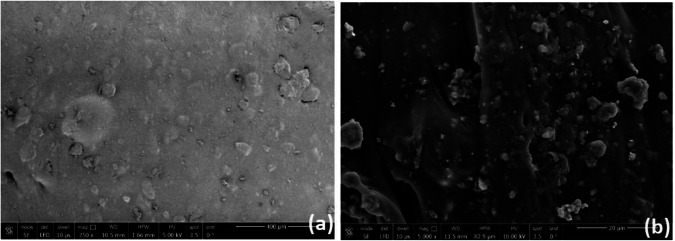
**a** S_1 FE-SEM scan with magnification 2500ˣ, **b** S_1 FE-SEM scan with magnification 10000ˣ

**Fig. 4:**
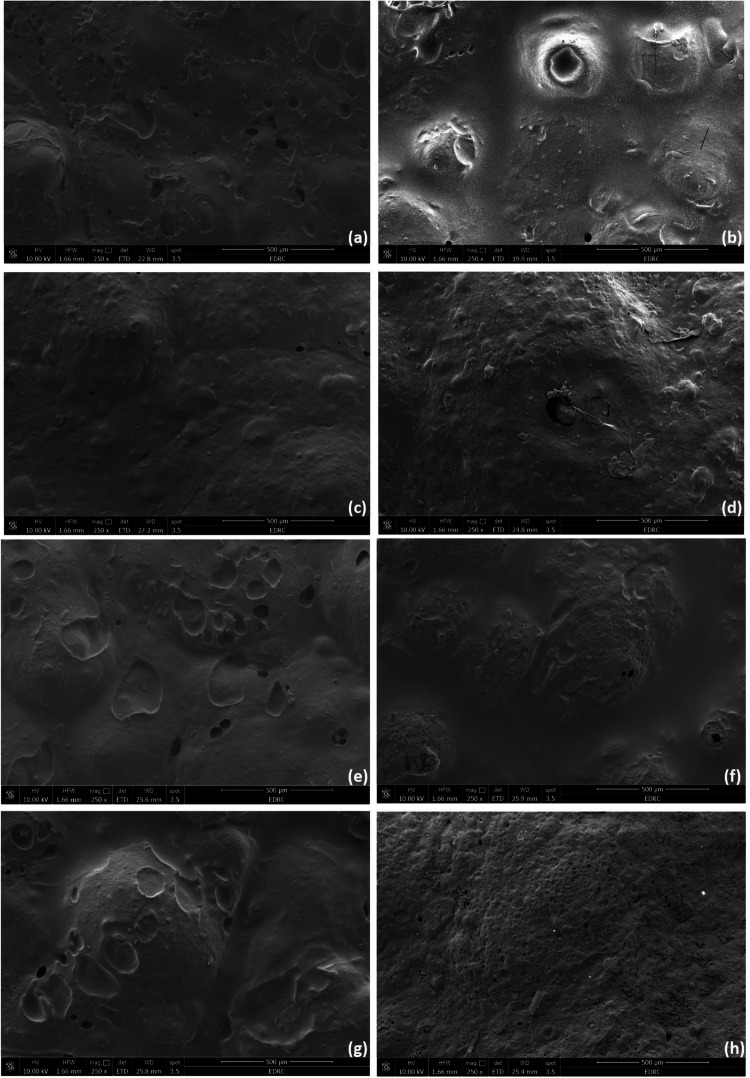
FE-SEM scan with magnification 250ˣ for cell adhesion to bone cement surface **a** S_2, **c** S_3, **e** S_4 and **g** S_5 after 1 day. FE-SEM scan with magnification 250ˣ **b** S_2, **d** S_3, **f** S_4 and **h** S_5 after 7day

The surface structure of pure bone cement is checked by FE-SEM scan in two magnifications as shown in Fig. 3. Figure 3a shows the 2500* magnification and Fig. 3b shows the 10000ˣ magnification of S_1 that clarifies an acceptable rough surface that could be attached to human bone. The addition of nanomaterials in modified bone cement shows a remarkable slight decrease in roughness as shown in Fig. 4. Modified S_2 surface scanned after one and seven days showed a non-homogenous surface scan with multi-pores. Modified S_3, S_4, and S_5 surface scans after one and seven days showed a more homogenous surface with a lower number of pores, especially for S_3 which contains the highest percentage of Ca_3_(PO_4_)_2_ NPs that showed several white dots indicating the embarkation of calcium in the specimen surface, this may lead to good attachment with the human bone after the impress of modified bone cement in the human body.

**Fig. 5:**
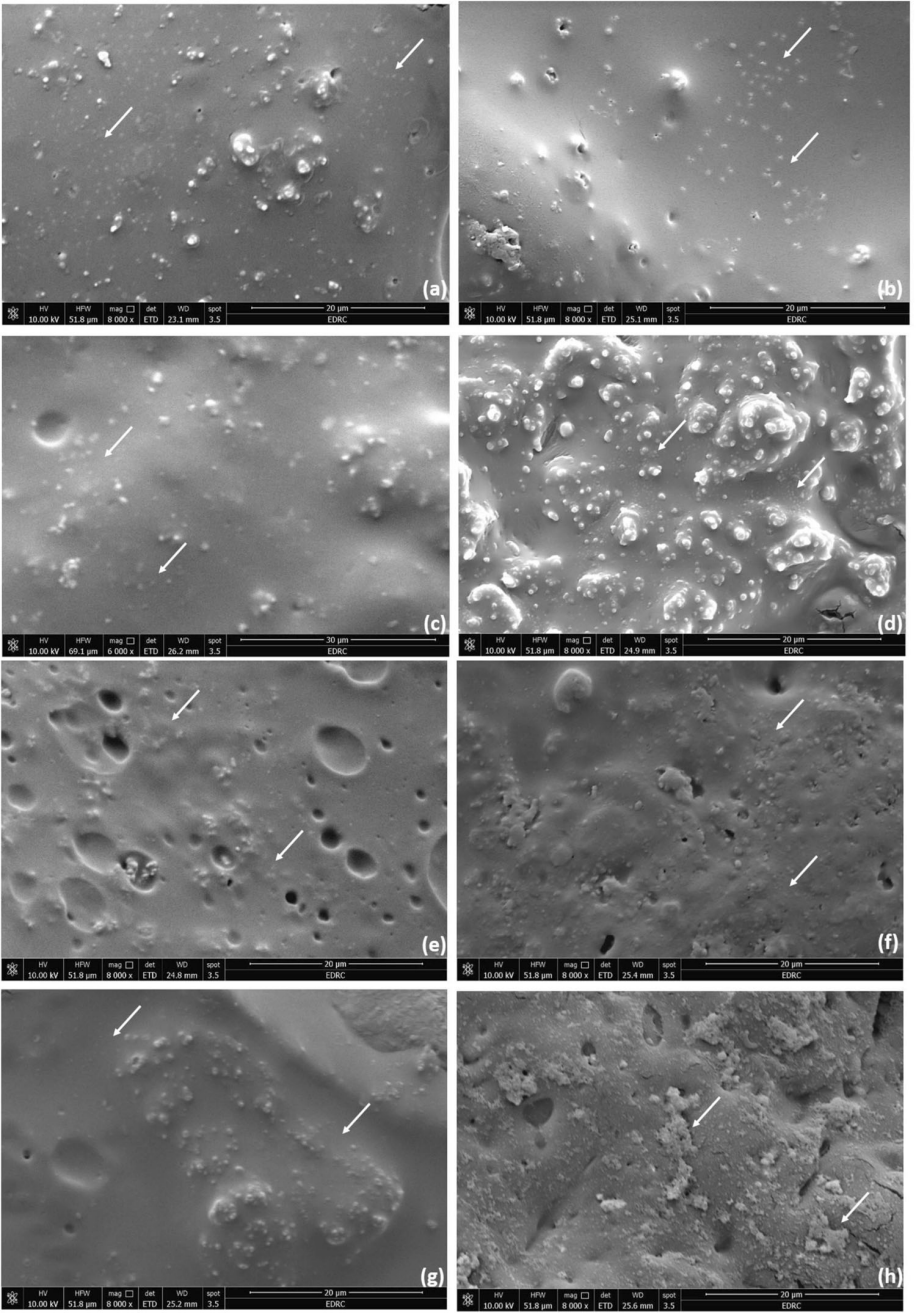
FE-SEM scan with magnification 8000ˣ for cell adhesion to bone cement surface **a** S_2, **c** S_3, **e** S_4 and **g** S_5 after 1 day. FE-SEM scan with magnification 8000ˣ **b** S_2, **d** S_3, **f** S_4 and **h** S_5 after 7day

The SEM micrograph indicates that MG63 cells were spread over the modified bone cement specimens as clarified in Figs. 4 and 5. Figure 4 shows the specimens’ surface with a magnification of 250 ˣ after 1 and 7 days of plenty MG63 osteoblast-like cells on specimens’ surface, and Fig. 5 shows the specimens’ surface with a magnification of 8000 ˣ after 1 and 7 days of plenty MG63 osteoblast-like cells on specimens’ surface. The FE-SEM scan of specimens after 1 and 7 days revealed the same shape of MG63 in bone cement specimens’ surface indicating successful cell adhesion, as illustrate with white sign in images. The FE-SEM scan of S_2 after 7 days which is shown in Fig. 5b revealed the same MG63 cell adhesion as the FE-SEM scan after 1 day as clarified in Fig. 5a. The FE-SEM scan of S_3 after 7 days is shown in Fig. 5d revealed many white dots compared to that appeared in the FE-SEM scan after 1 day as shown in Fig. 5c, which clarifies the effect of adding 2% of Ca_3_(PO_4_)_2_ NPs to specimens. The FE-SEM scan of S_4 after 7 days is shown in Fig. 5f showed revealed the same MG63 cell adhesion as the FE-SEM scan after 1 day as clarified in Fig. 5e. The FE-SEM scan of S_5 after 7 days is shown in Fig. 5h revealed several MG63 lines more than that appeared in the FE-SEM scan after 1 day as clarified in Fig. 4g which identifies cell growth.

**Fig. 6:**
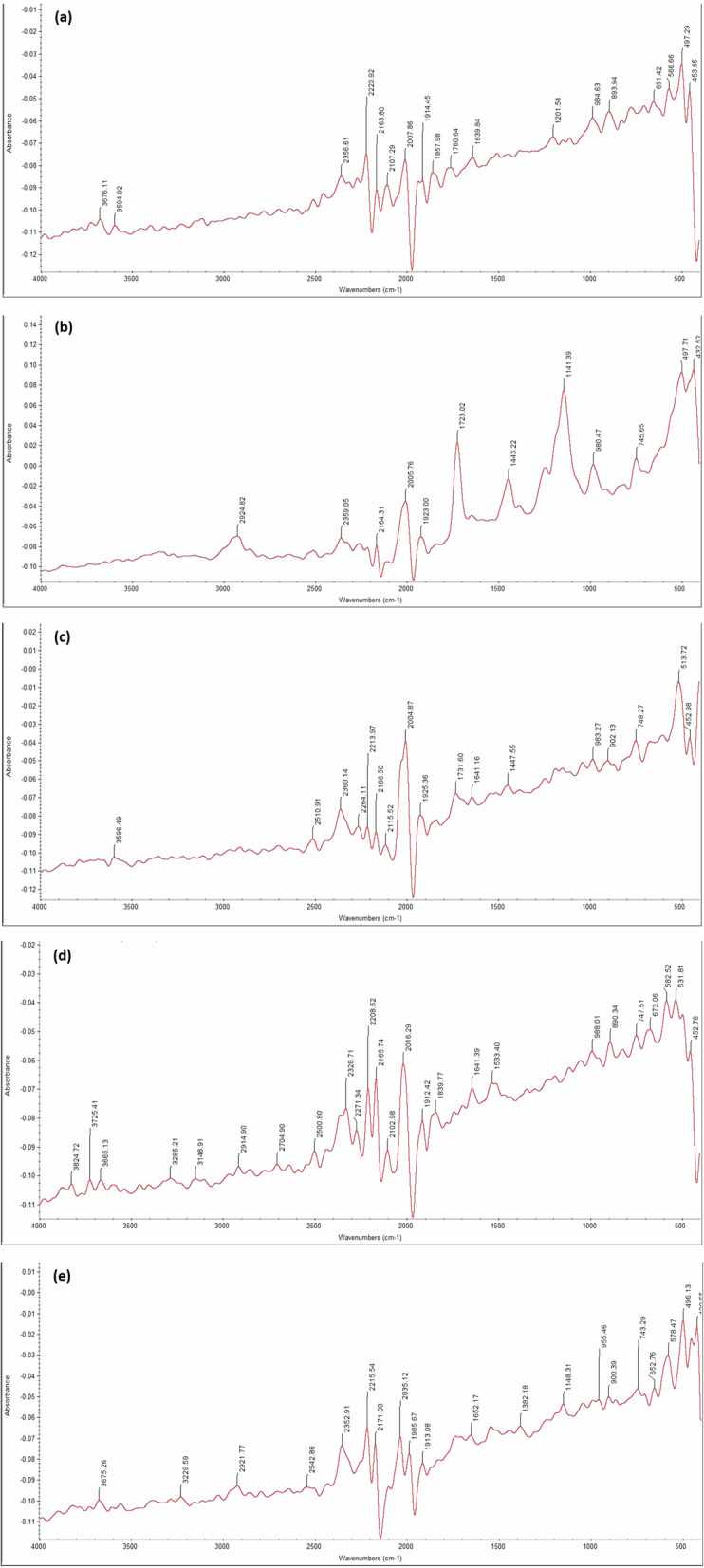
FTIR absorption of different five samples **a** S_1, **b** S_2, **c** S_3, **d** S_4 and **e** S_5

**Fig. 7:**
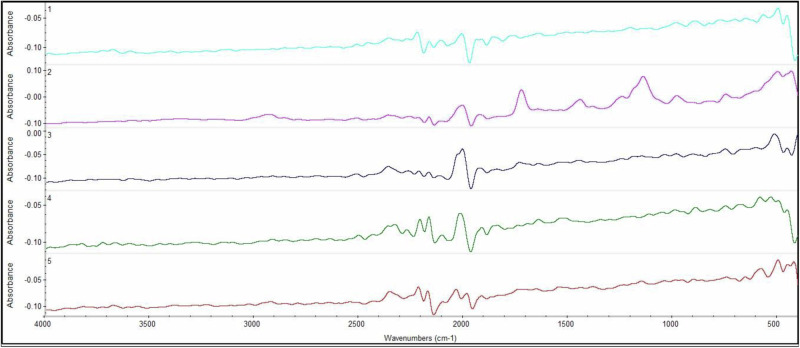
The combination of FTIR absorption of the experimented five specimens

FTIR of the experimented five specimens is clarified in Fig. 6 using wavelength range from 4000 to 400 cm^−^^1^. S_1 FTIR is illuminated in Fig. 6a, and specimen S_2 FTIR is clarified in Fig. 6b. Modified specimens S_3, S_4, and S_5 FTIR are shown in Fig. 6c, d, and e respectively. Results clarify that the peaks of pure bone cement are correspondent with the main chemical bonding of bone cement [40]. Modified specimens’ primary chemical bonding peaks correspond to that of the pure bone cement, except for the additional peaks that are created owing to the additive materials. The five specimens’ FTIR absorption curves are shown in Fig. 7 which clarifies the convergence between modified specimens with the pure, which indicates preserving all specimen components during polymerization.

**Fig. 8:**
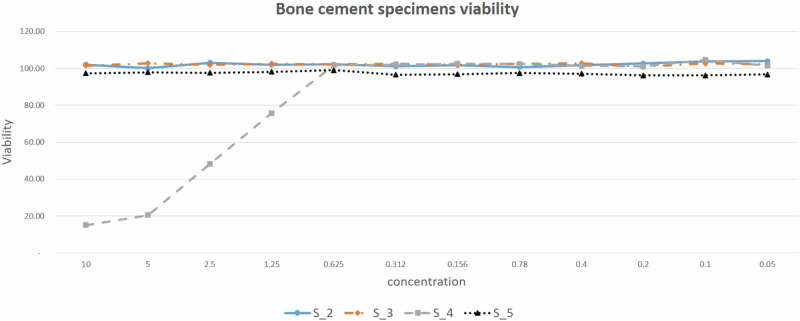
Toxicity test results through the evaluation of viability and the concentration mg/ml using MTT assay

Cell toxicity or MG63 cell viability of pure and modified bone cement specimens has been examined through MTT assay as shown in Fig. 8. The viability of the MG63 cells seeded on the specimen surface was taken as a control, cell viability was found to be 100% for S_2, S_3, and S_5 bone cement specimens, where all of the MTT solution changed to violet color which means no toxic change in human cells. However, the absolute value of cell proliferation among S_4 samples was decreased by 1.25 mg with the increase of Al_2_O_3_ NPs.

### Physical properties results

In the practical use of bone cement, the temperature value during polymerization has an important effect in indicating the operation’s success, because of the nerves and tissues that are affected by temperature. Setting temperature indicates the acceptable bone cement polymerization temperature value. Recorded setting temperature values are 39.58, 41.63, 41.3, 41.53, and 39.98 °C for S_1, S_2, S_3, S_4, and S_5 respectively as shown in Fig. 10a.

Porosity has an indirect relation with mechanical properties, but in bone cement, it may be important to get with limited porosity percentage to allow for the growth and formation of bone cells. The maximum porosity percentage value is 22.51% recorded for S_5, 18.82% for S_4, 15.58% for S_1, and 15.98% for S_2. The minimum porosity percentage value is 10.41% for S_3. Figure 10b clarifies the porosity values of the experimented five specimens.

**Fig. 9:**
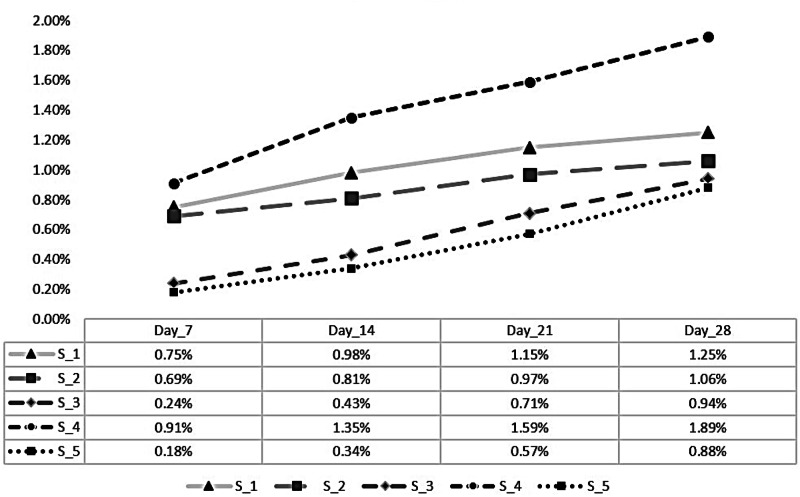
Degradation of the five specimens after 0, 7, 14, 21 and 28 days

Degradation affects negatively the durability of bone cement and, thus should be avoided or limited. Bone cement should be biodegradable to achieve its main job of fixing prosthetic joints. Limited degradation values keep a long fixation lifetime. Figure 9 clarifies the degradation percentage after 7, 14, 21, and 28 days for pure and modified bone cement specimens. Results clarified that S_2, S_3 and S_5 recorded lower degradation values of 1.06%, 0.94% and 0.88%, lower than pure bone cement S_1 which recorded 1.25% after 28 days. S_4 recorded maximum degradation value 1.89% over all tested specimens.

### Mechanical properties results

**Fig. 10:**
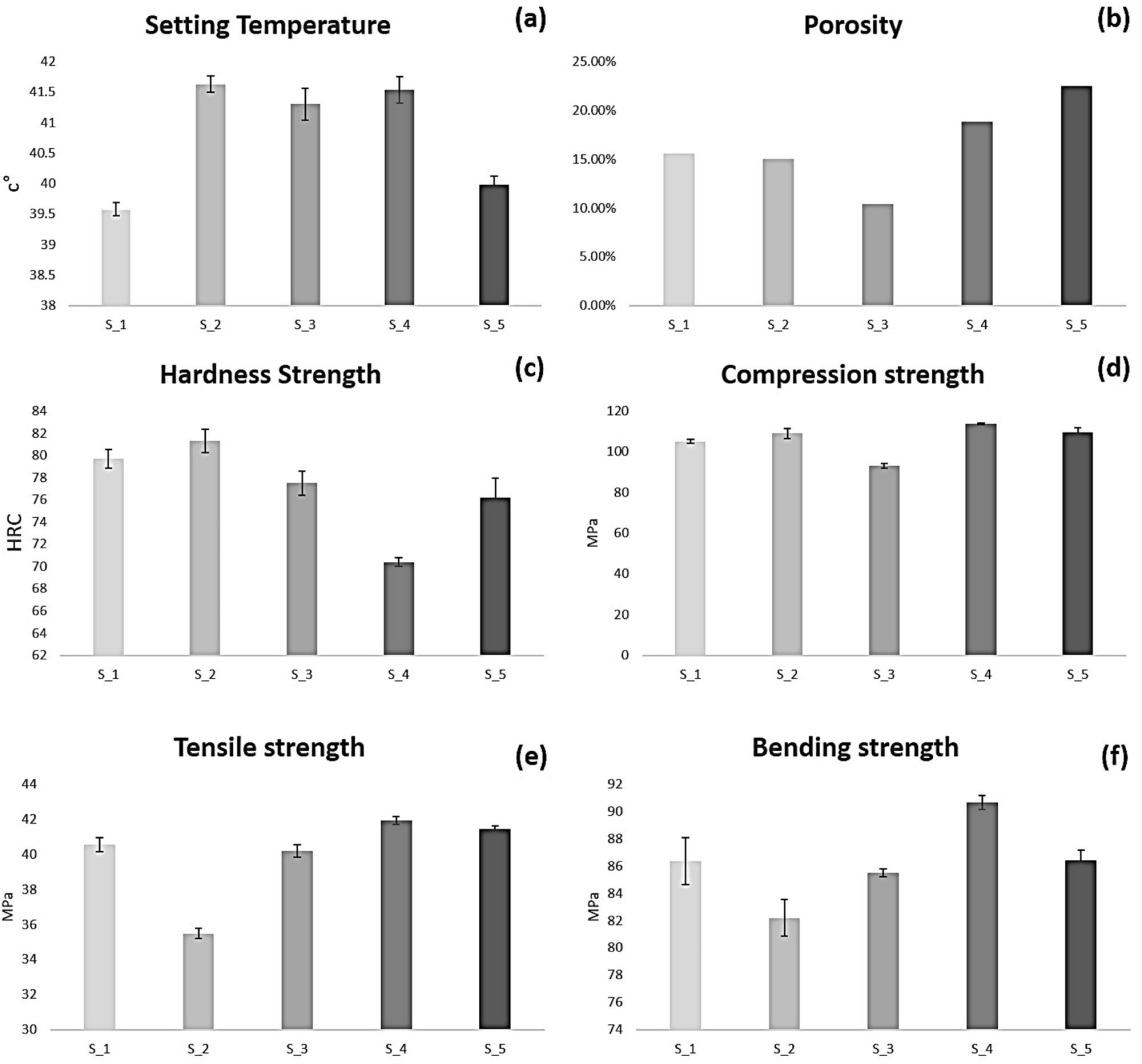
**a** Setting temperature values for the experimented 5 specimens, **b** Porosity percentage, **c** Hardness values, **d** Compression strengths, **e** Tensile strengths, **f** Bending strengths

Hardness of bone cement indicates the ability to endure surface scratch. Figure 10c clarifies the Vickers micro-hardness test values of the five specimens, results clarified that pure bone cement hardness recorded 79.7 HRC. Specimen S_2 recorded maximum hardness 81.3 HRC. Modified bone cement specimens S_3, S_4 and S_5 recorded 77.5, 70.4 and 76.6 HRC respectively.

Compression strength value plays a main role in restricting the bone cement loading capacity. It recorded 105.05 MPa for pure bone cement and 108.98 MPa for specimen S_2. Modified bone cement specimens recorded conflicting strength values, S_4 and S_5 showed noticeable improvement recording 113.6 and 109.4 MPa, but S_3 showed noticeable decrease recording 92.99 MPa. Figure 10d shows S_1, S_2, S_3, S_4 and S_5 compression strength values.

Figure 10e shows the tensile strength of pure and the three modified bone cement specimens. Results clarify that the maximum tensile strength is recorded with the modified bone cement S_4 and the minimum with the bone cement specimen S_2. The tensile strength test recorded 40.54, 35.47, 40.19, 41.92 and 41.44 MPa for S_1, S_2, S_3, S_4 and S_5 respectively.

Bending strength test clarifies the maximum load of deflection before specimen rupture, and it has direct relation with the performance of bone cement mechanical properties. Figure 10f shows the average bending strength of the three samples for each experimented bone cement specimen. Bending strength recorded 86.35, 82.19, 85.5, 90.63 and 86.39 MPa for S_1, S_2, s_3, S_4 and S_5 respectively.

## Discussion

Ideal bone cement specifications require not only strong mechanical properties like compression and bending strengths but also biocompatibility, osteoblasts, non-degradability, non-toxicity, and acceptable physical properties that are medically compatible with the human body. As clarified in the results, modified bone cement specimens provide important enhancement in biological, physical, and mechanical properties compared to the natural human bone properties as clarified in Table 2. MgO and Ca_3_(PO_4_)_2_ NPs have a direct influence on the biological and physical aspects of bone cement because they aid in maintaining good adhesion to normal bone. This is clarified with good cell adhesion and the provided acceptable physical properties. Al_2_O_3_ and TiO_2_ NPs have a direct effect on enhancing mechanical properties, relative to the provided enhancement in compression, tensile, and bending strengths [41].

**Table 2:** Conclusion of the specimen tests properties compared to cortical bone

Test	Hardness strength (MPa)	Compression strength (MPa)	Tensile strength (MPa)	Bending strength (MPa)	Setting temperature (C°)	Degradation % 28 days	Porosity %
**cortical bone ranges** [42–48]	78–151	90 to 230	50–151	103–238	37 C°	-	Average 3.5%
S_1	79.7	105.05	40.54	86.35	39.58	1.25%	15.58%
S_2	81.3	108.98	35.47	82.19	41.63	1.06%	14.98%
S_3	77.5	92.99	40.19	85.5	41.3	0.94%	10.41%
S_4	70.4	113.6	41.92	90.63	41.53	1.89%	18.82%
S_5	76.2	109.4	41.44	86.39	39.98	0.88%	22.51%

Biological enhancement is tested by introducing osteoblast-like-cells MG63 cells to modified bone cement specimens to measure cell toxicity using MTT assay and cell adhesion using FE-SEM. In the modified specimen containing MgO and Al_2_O_3_ NPs only, the FE-SEM scan showed weak cell adhesion compared to other modified specimens with low and far spaces between MG63 cells at the specimen surface, and abnormal MG63 cells with many pores. Ca_3_(PO_4_)_2_ NPs additive material revealed an admirable cell adhesion performance because MG63 osteoblast-like cells show cytoplasmic extension with it, and filopodia can be observed on the MG63 osteoblast-like cells through FE-SEM scan of the modified specimen that includes 1%, 1.5 and 2% of Ca_3_(PO_4_)_2_ w/w NPs. Especially, the modified bone cement specimens that contain 0.5% MgO, 1.5% Ca_3_(PO_4_)_2_, 1.5% Al_2_O_3,_ 1.5% TiO_2_ NPs, and 0.5% MgO, 1% Ca_3_(PO_4_)_2_, 2% Al_2_O_3,_ and 1.5% TiO_2_ NPs demonstrated robust cell growth that visually appeared in FE-SEM scan image, where high and near spaces between MG63 cells are observed at specimen surface. Cell viability test demonstrates that all additive MgO, Ca_3_(PO_4_)_2_, Al_2_O_3,_ and TiO_2_ NPs specimens are completely nontoxic, except specimens that contain 0.5% of MgO, 1% of Ca_3_(PO_4_)_2_, 2% of Al_2_O_3,_ and 1.5% of TiO_2_ NPs that recorded 25% of toxicity that can be avoided using suitable antibiotics.

FTIR spectrometer technique analysis identifies the polymer groups of modified bone cement specimens, in addition to the new peaks that appear due to adding MgO, Ca_3_(PO_4_)_2_, Al_2_O_3,_ and TiO_2_ NPs to pure bone cement.

Using all of the nano additive materials of magnesium, titanium, calcium, and aluminum with oxide to pure bone cement showed approximately no toxicity in any of the experimented specimens. Applying the MTT assay toxicity test proves no toxicity in S_2, S_3, and S_5, but clarifies that there is a slight percentage of toxicity in S_4 which may be produced from the high percentage of Al_2_O_3._

Setting temperature for the modified bone cement specimens produced acceptable variable values, raised by 2 °C. The acceptable porosity range of bone cement is from 5 to 20%, all of the specimens showed normal porosity range except S_5 which produced a 22% porosity value. Degradation of modified bone cement specimens decreased by up to 0.37%, except S_4 which had a 0.64% increase in degradation percentage.

Generally, all of the modified bone cement specimens showed at least one enhancement in the tested mechanical properties. The hardness of the modified specimen that contain TiO_2_ and MgO is raised by 1.6 HRC, whereas the modified specimens that contain Ca_3_(PO_4_)_2_ and Al_2_O_3_ showed a decrease in hardness value with 2 HRC.

The effect of adding Al_2_O_3_ and TiO_2_ NPs in enhancing the compression strength is common dissection, modified bone cement specimens showed remarkable improvement up to 8.55 MPa, except S_3 which contains 2% Ca_3_(PO_4_)_2_ that showed a decrease in the compression strength by 12.6 MPa.

Tensile strength for the modified specimen that contains TiO_2_ and MgO decreased with an acceptable value for bone cement tensile strength range, but in modified bone cement with adding Ca_3_(PO_4_)_2_ and Al_2_O_3_ it recorded enhancement by 1.38 MPa.

The bending strength test simulates the effect of human motion and it is a key factor in choosing the suitable bone cement with additive ratios, especially when used in prosthetic joint fixation such as the prosthetic hip joint. S_2 and S_3 recorded a decrease in bending strength values by up to 4.16 MPa. S_4 recorded a high value of bending strength enhancement by 4.28 MPa. Table 2 summarizes the findings of all tests in comparison to the normal cortical bone range.

Thus, the modified specimen S_4 with 95% pure bone cement powder, 0.5% MgO, 1.5% TiO_2_, 1% Ca_3_(PO_4_)_2_ and 2% Al_2_O_3_ NPs is recommended for use with the suitable antibiotics in prosthetic fixation operations with patients over 60 years This is due to its remarkable improved compression strength by 8.13%, tensile by 3.4% and bending by 4.95% that leads to better stress distribution, high load bearing and loosening avoidance.

It is also recommended to use modified specimen S_5 with 95% pure bone cement powder, 0.5% MgO, 1.5% TiO_2_, 1.5% Ca_3_(PO_4_)_2_, and 1.5% Al_2_O_3_ NPs without antibiotics in prosthetic fixation operations for people having moderate cortical bone strength, due to its improved compression strength by 4.14%, tensile by 3.4%and bending by 0.18%.

## Conclusion

This study investigates the biological, physical, and mechanical properties of pure and four modified bone cement specimens. The first modified bone cement specimen includes 98% w/w of pure bone cement with 0.5% MgO and 1.5% TiO_2_ NPs. The other three modified bone cement specimens are made by 95% w/w of pure bone cement, 0.5% MgO, 1.5% TiO_2,_ and 3% various ratios of Ca_3_(PO_4_)_2_ and Al_2_O_3_ NPs. The results showed that all modified specimens had more homogeneous and smoother surfaces with the FE_SEM scan. Specimens with 1.5% or more Ca_3_(PO_4_)_2_ NPs exhibited better cell adhesion, while those with more than 1.5% Al_2_O_3_ NPs showed larger MG63 cells, indicating potential for good cell growth. FTIR analysis confirmed the presence of NPs in the modified specimens. Toxicity tests indicated that specimens with less than 2% Al_2_O_3_ NPs are safe for human use, while those with 2% Al_2_O_3_ showed slight 1.25% toxicity that can avoided by using suitable antibiotics. The setting temperature of modified specimens increased by 5.17%. High Ca_3_(PO_4_)_2_ content reduced porosity by 5.17%, whereas high Al_2_O_3_ content increased porosity by 6.93%. Modified specimens had reduced hardness, especially those with equal ratios of Al_2_O_3_ and Ca_3_(PO_4_)_2_, which decreased to 9.4 HRC. Compression strength improved by 8.55 MPa in specimens with 2% Al_2_O_3_ NPs. Tensile strength slightly increased by 1.38 MPa in modified specimens, and bending strength improved by 4.28 MPa with equal ratios of Al_2_O_3_ and Ca_3_(PO_4_)_2_.

Area of future work could include applying in-vitro and in-vivo experiments for all modified bone cement specimens containing Ca_3_(PO_4_)_2_ and Al_2_O_3_ NPs such as measuring the cell growth. It is also recommended to use a tool for simulating the body motion effect on prosthetic joints fixed by modified bone cement to check for the enhanced properties under normal working conditions.

## Supplementary information

Data supplementary

Data supplementary

## Data Availability

The datasets used and analyzed during the current study are available from the corresponding author upon reasonable request.
