# Supplementary material for: Enhanced bone cement for fixation of prosthetic joint utilizing nanoparticles
Source: J Mater Sci Mater Med. 2025 Jan 13;36(1):10. doi: 10.1007/s10856-024-06848-1 (PMC11729110; doi:10.1007/s10856-024-06848-1)

**Data supplementary**

- 1. **Field emission scanning electron microscopy (FE-SEM) scan**

The pure bone cement specimen coated by gold to allow scanning by FE-SEM.


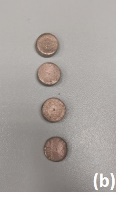


The composite and modified specimens that had osteosarcoma cells on the surface with one and seven days


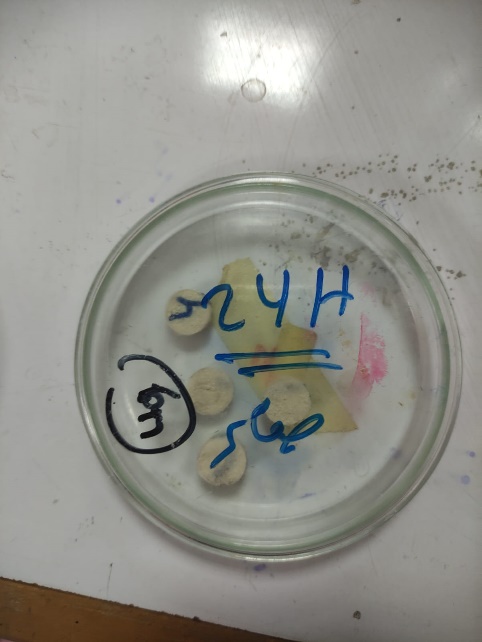

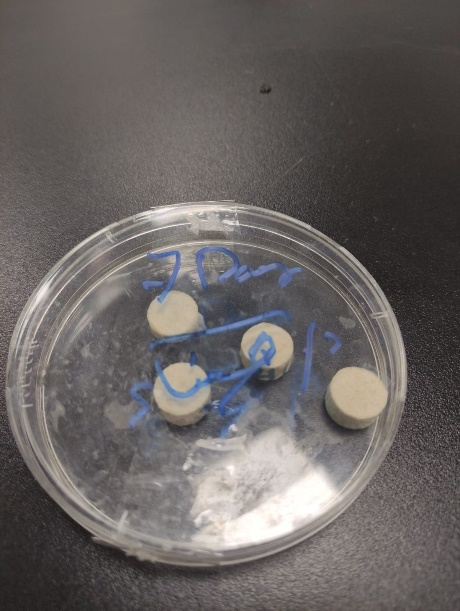


The specimens in holder before and after coated with gold to allow FE-SEM scan


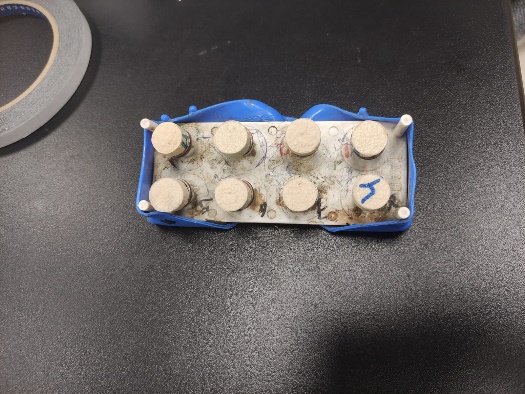

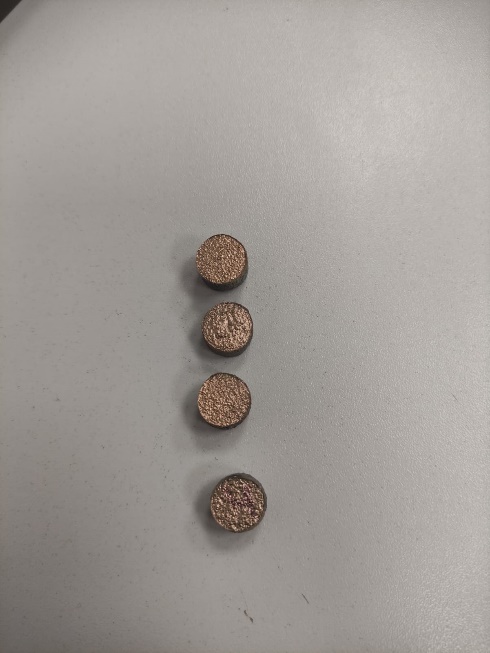


The specimens in FE-SEM (QUANTA FEG 250) device holder to scan


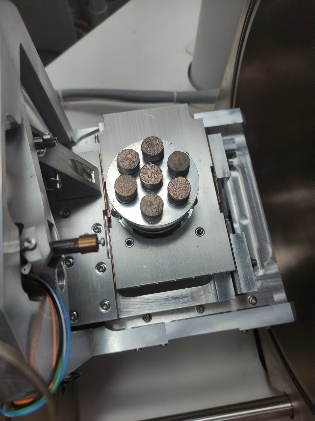

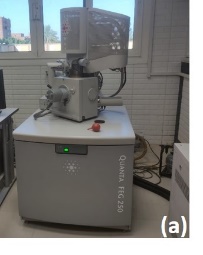


- 1. **Setting temperature**

The setting temperature was measured using thermocouple with type E that impressed to the center of bone cement specimens.


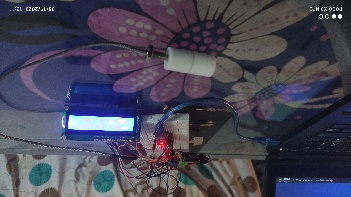

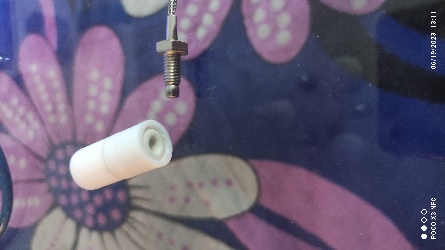


- 1. **Porosity and Degradation**

The porosity and degradation were calculated using marked beakers, incubators and sensitive balance with 4-digit accuracy.


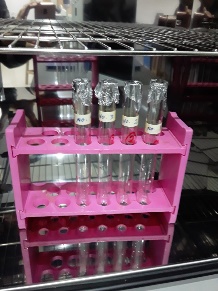

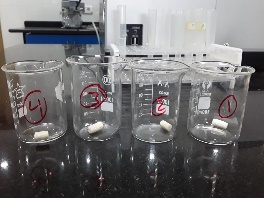


- 1. **Hardness test**

The Vickers micro-hardness device that used to measure the hardness and the pure specimen after measured.


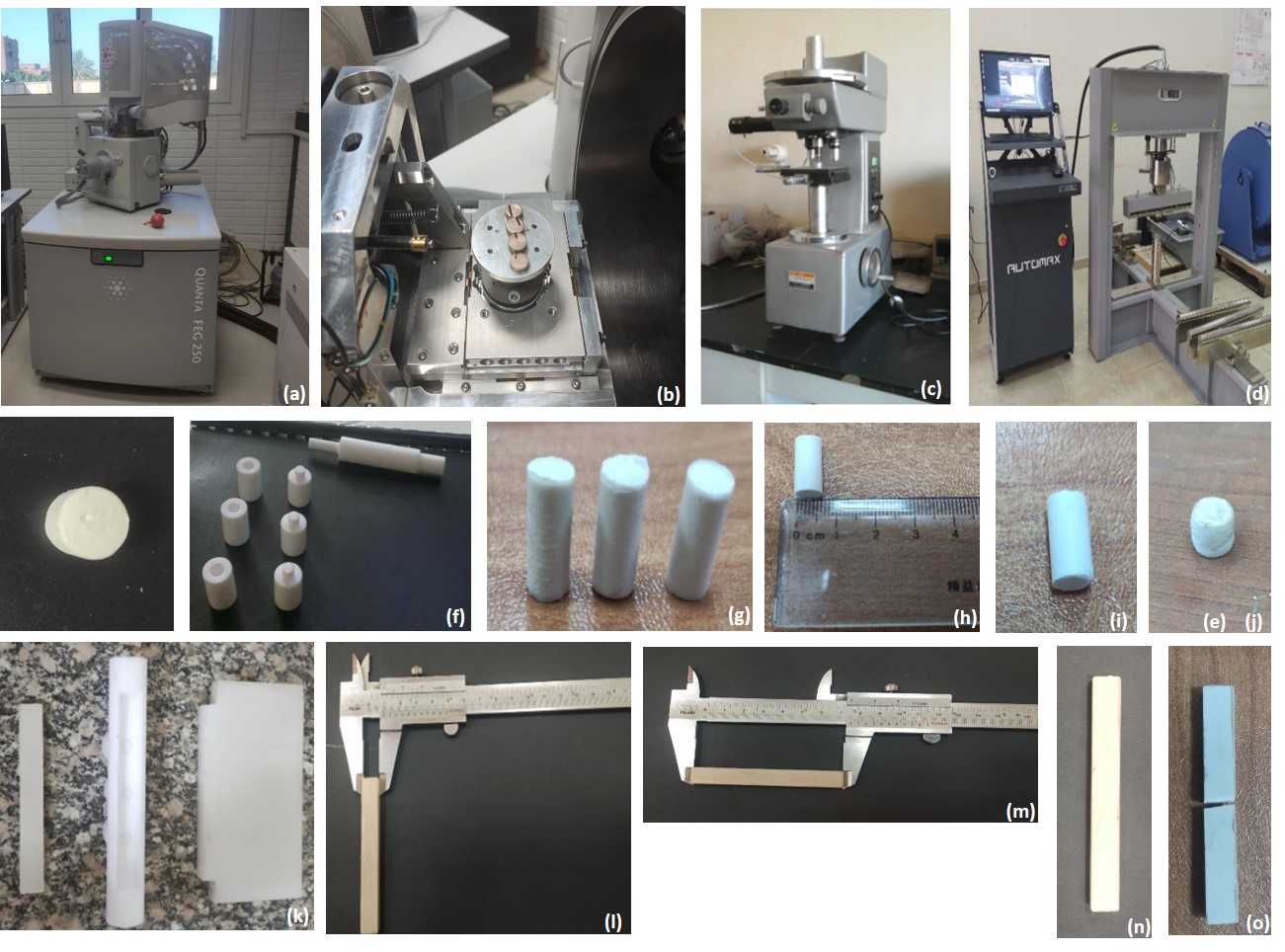

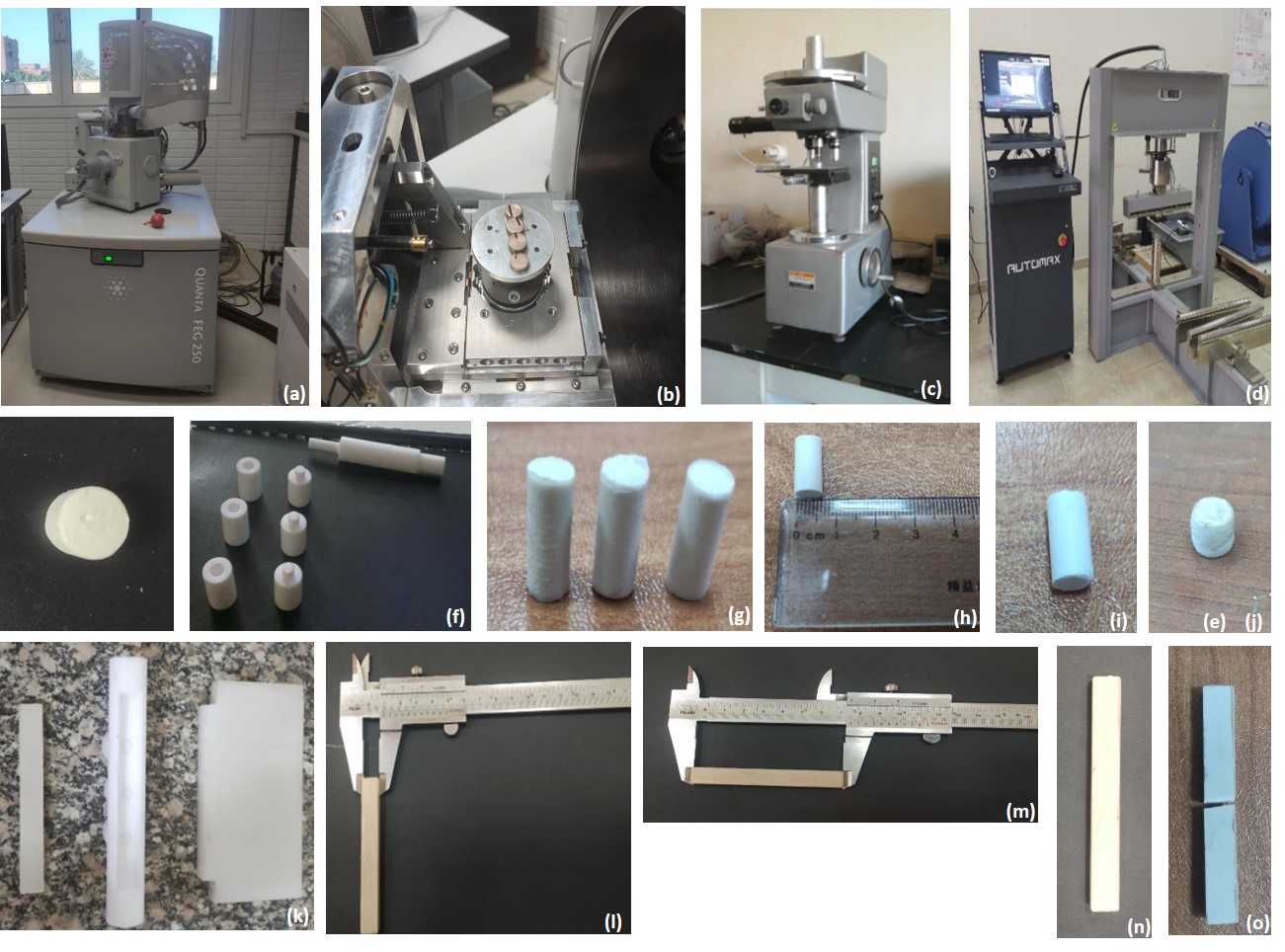


- 1. **Compression strength test**

The Pure bone cement compression specimens dimension


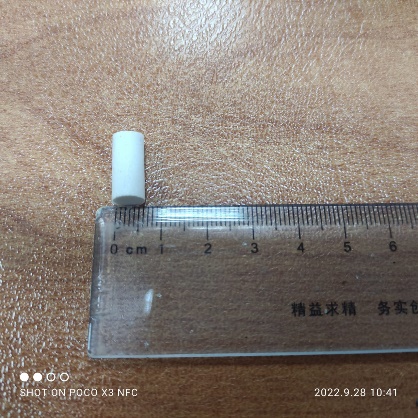

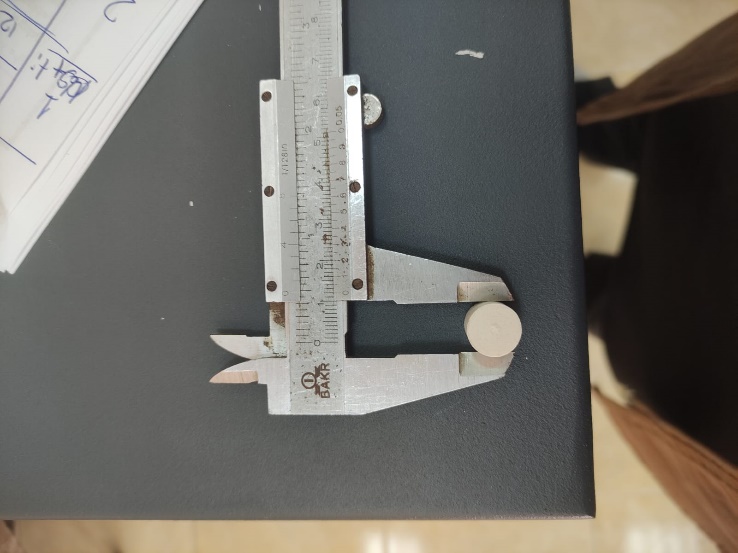

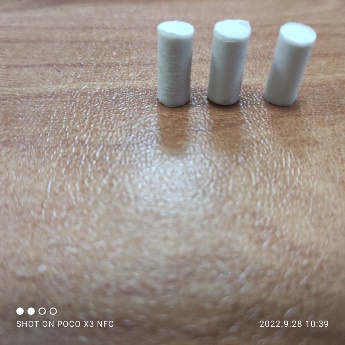


The Pure bone cement specimen before and after compressed


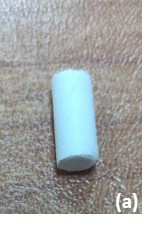

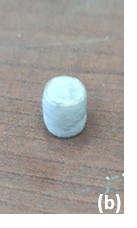


The AUTOMAX MULTITEST computerized control machine to measure compression strength


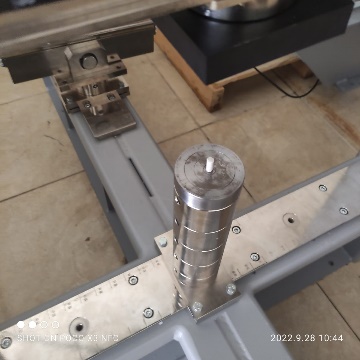

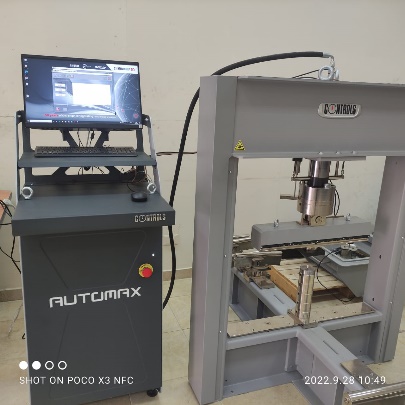


- 1. **Tensile strength test (diametrical compression)**

The Pure bone cement tensile specimen dimension


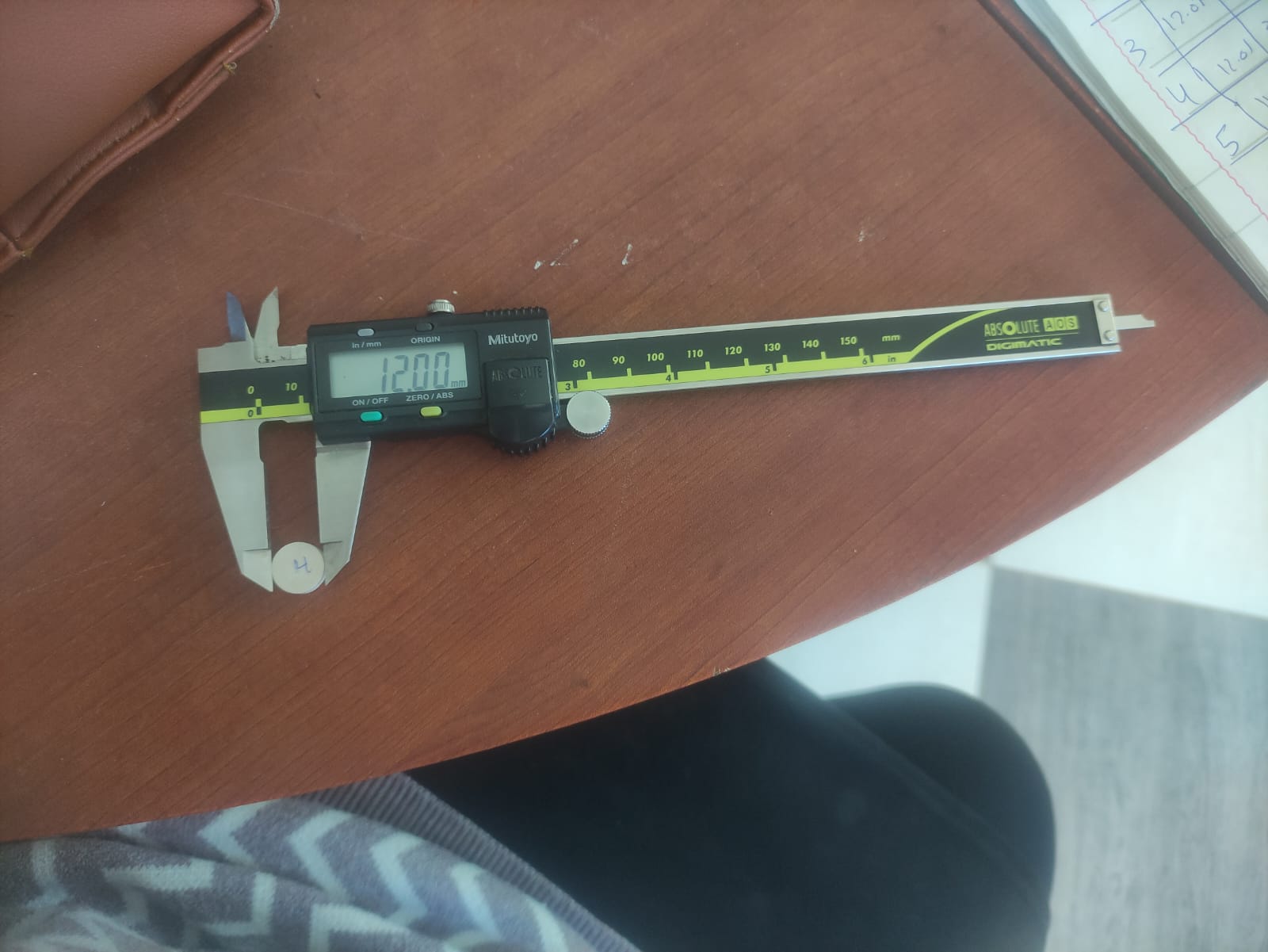


The Pure bone cement specimen before and after compressed


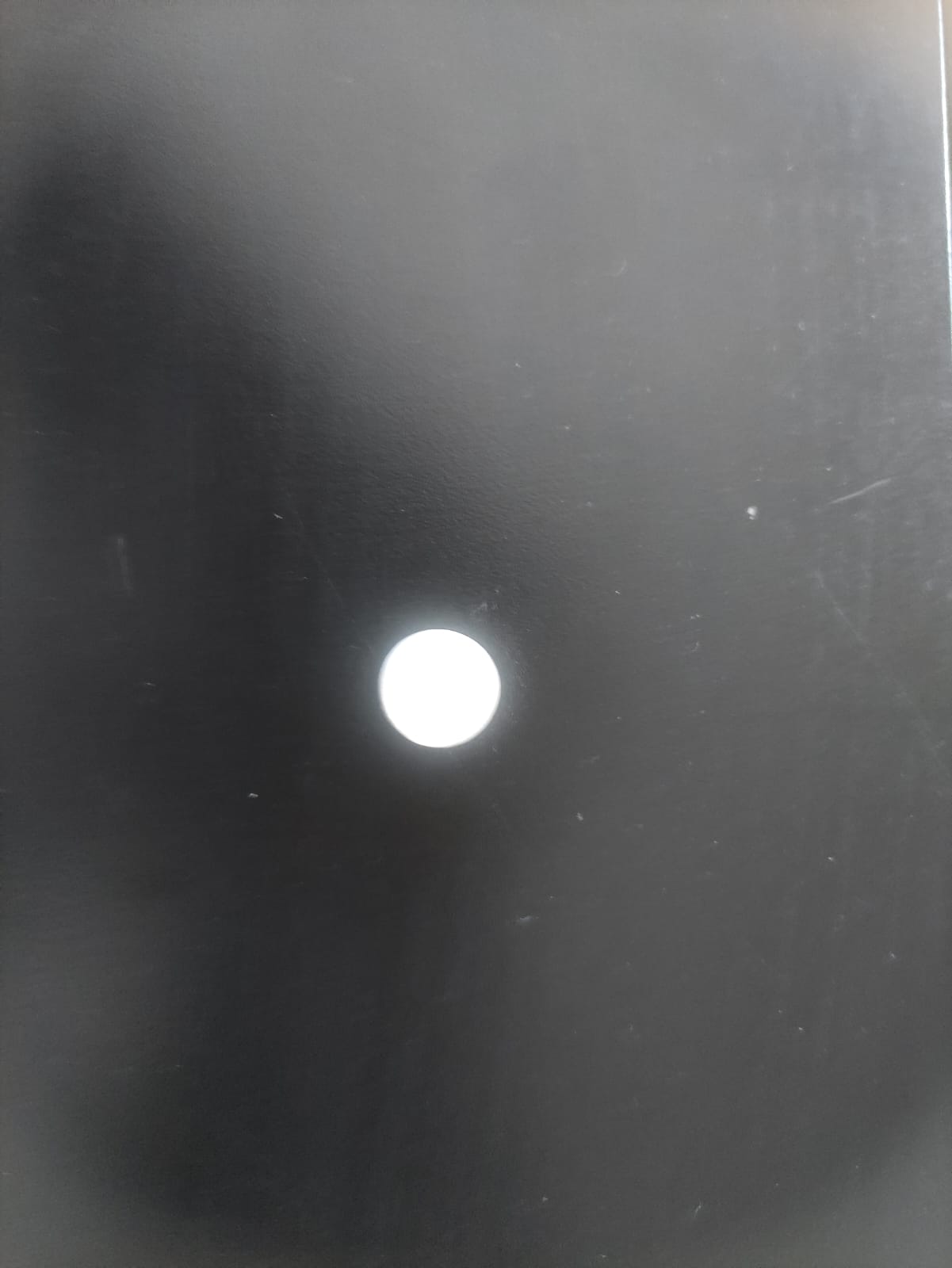

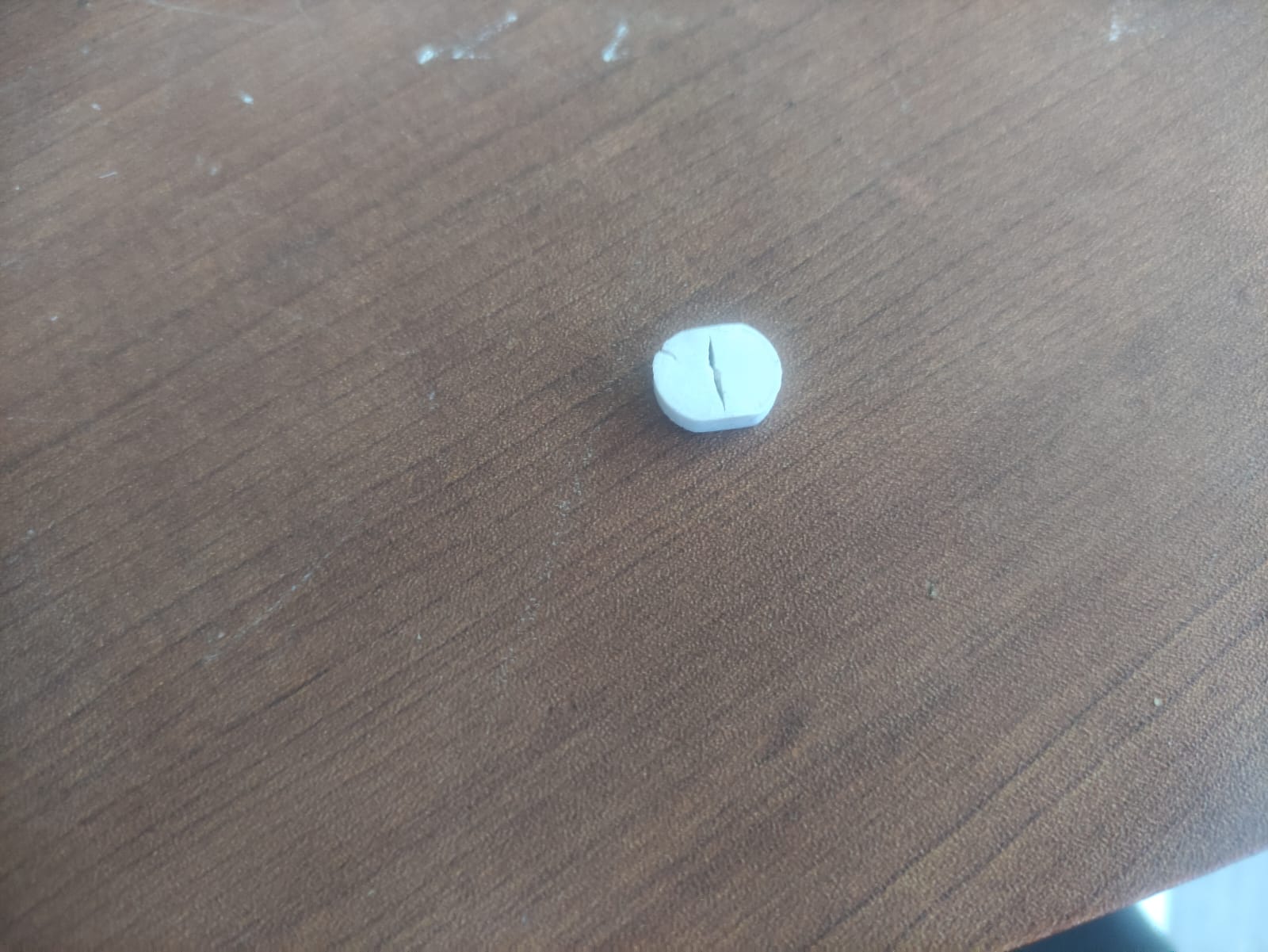


The universal test machine to measure diametrical compression


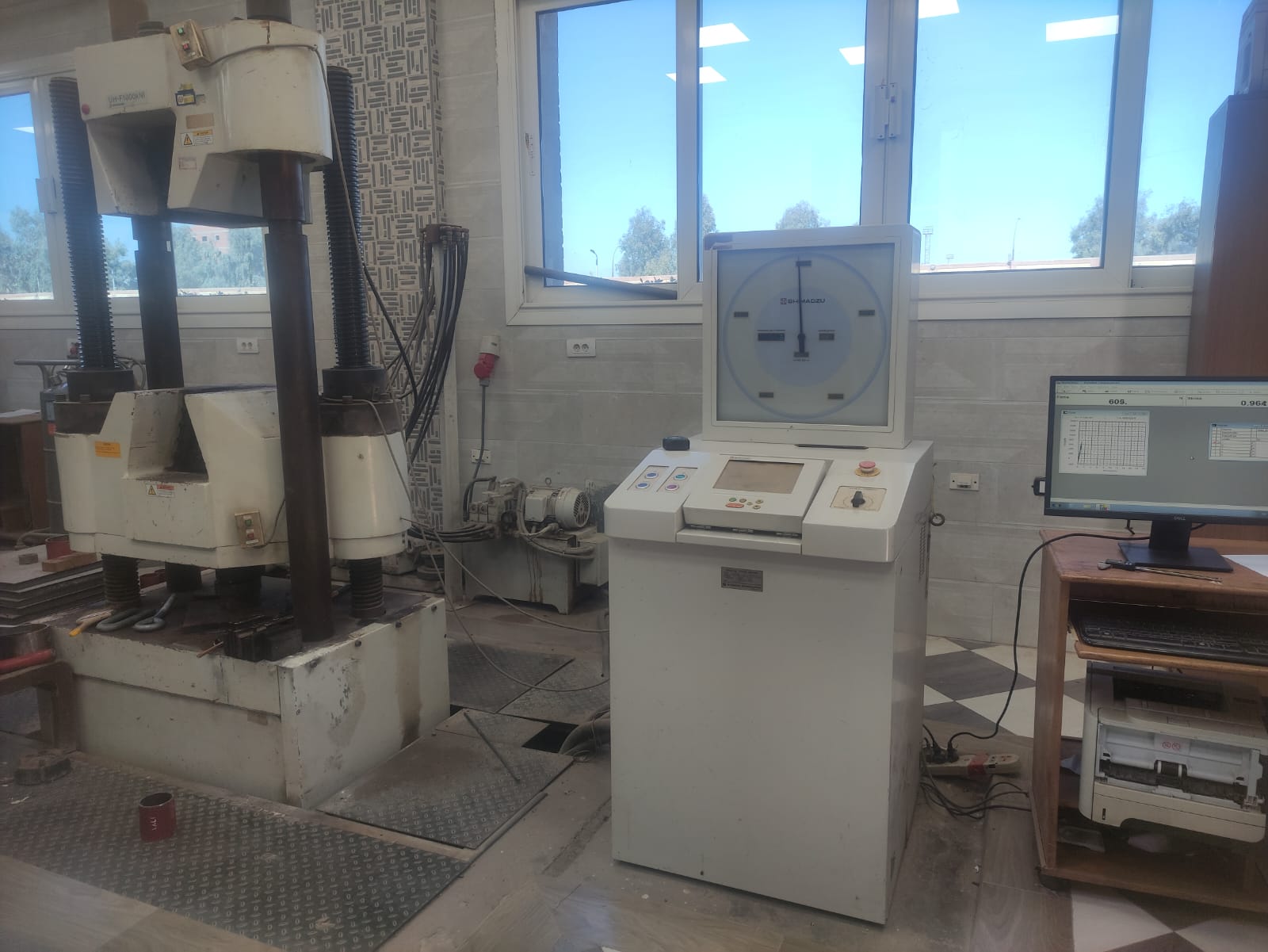

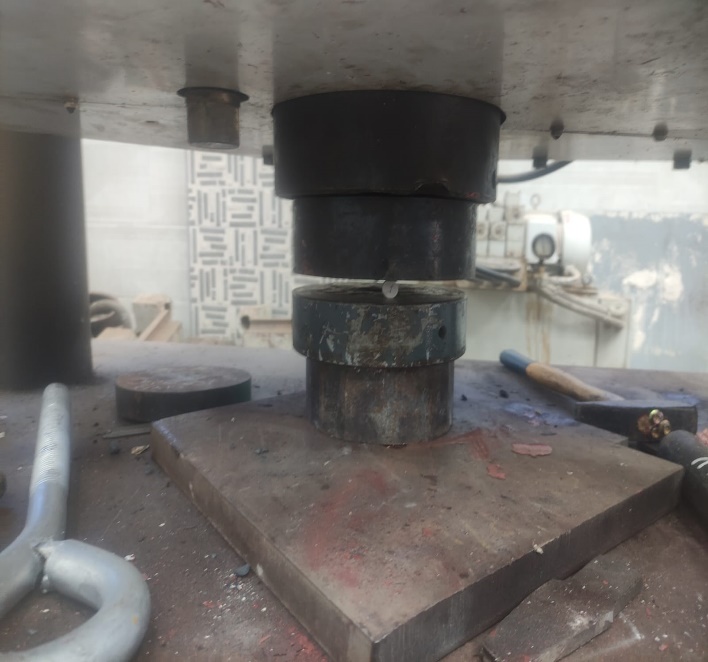


- 1. **Bending strength test**

The Pure bone cement bending specimen dimension


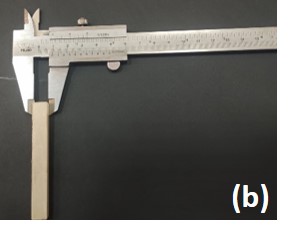

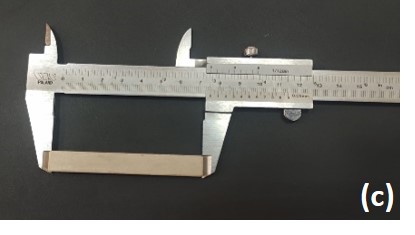


The pure bone cement bending specimen mold


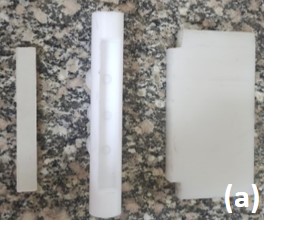


The smoothing machine before bending


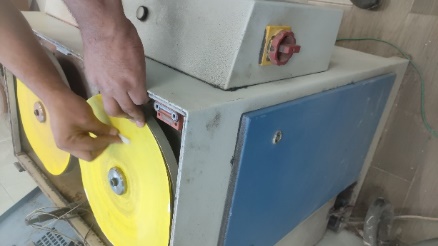

Supplement: Supplementary file 1 — Data supplementary [file 10856_2024_6848_MOESM1_ESM.docx]
